# Anti-Tumor Effects of Vitamin B2, B6 and B9 in Promonocytic Lymphoma Cells

**DOI:** 10.3390/ijms20153763

**Published:** 2019-08-01

**Authors:** Kathleen Mikkelsen, Monica D. Prakash, Nyanbol Kuol, Kulmira Nurgali, Lily Stojanovska, Vasso Apostolopoulos

**Affiliations:** 1 Institute for Health and Sport, Victoria University, Werribee Campus, Melbourne, VIC 3030, Australia; 2 School of Health and Biomedical Sciences, RMIT University, Bundoora Campus, Melbourne, VIC 3083, Australia; 3 College of Food and Agriculture, Department of Nutrition and Health, United Arab Emirates University, Al Ain 16427, UAE

**Keywords:** vitamin B complex, pro-monocytes, U937 cell line, riboflavin, pyridoxine, folate, vitamin B2, vitamin B6, vitamin B9

## Abstract

Chronic inflammation can lead to tumour initiation and progression. Vitamin B complex has the ability to regulate the immune response and, therefore, inflammation but many of the mechanistic and molecular processes involved in this regulation are still not fully understood. This study sought to determine some of these processes by studying the effects of vitamin B2 (riboflavin) B6 (pyridoxine) and B9 (folic acid) on un-differentiated pro-monocytic lymphoma cells in regard to their ability to alter the proliferation, migration, apoptosis, cytokines and expression levels of programmed death ligand 1. We show that vitamin B2, B6 and B9, on pro-monocytic lymphoma cells exerted an anti-tumorigenic effect. This data could form the basis for future studies in using vitamin B supplementation to reduce cancer cell growth in vivo.

## 1. Introduction

Chronic inflammation can lead to cancer via epigenetic change. Infection and irritation instigating chronic inflammatory states, can produce environments that promote genomic lesions and tumor initiation, activate oncogenes and cause dysfunction of tumor suppressors leading to cancer development [1]. Genetic change as a result of inflammation effectively provides cancer cells with a mechanistic means of survival. Current scientific thought on inflammation and cancer progression centres on finding a means to dampen inflammation in order to prevent cancer development and provide a possible means of treatment [2].

There is a clear association between inflammation and cancer development demonstrated by numerous clinical and epidemiological studies. In fact, patients who suffer from inflammatory bowel disease such as ulcerative colitis and Chrohn’s disease are 10 times more likely to develop colorectal cancer [3,4,5], whilst cancers of the gastrointestinal tract, prostate and liver have been shown to originate from sites of chronic inflammation [6,7,8].

There are also a number of studies in the literature describing the role of vitamin B in the regulation of immune responses and inflammation [9,10,11,12,13]. Inadequate levels of certain B vitamins can drastically alter immune response by effecting the production of nucleic acid and protein synthesis, inhibiting the activity of immune cells and interfering with metabolic processes including methylation, serine, glycine and purine cycles. Inefficient methylation can lead to hyperhomocysteinemia which causes systemic and vascular inflammation contributing to the pathophysiology of other diseases [14,15,16,17]. Many of the mechanistic effects of vitamin B deficiency correlate with the properties of cancer development.

Current knowledge of B vitamins and inflammation predominantly revolves around the systemic effects of vitamin B in deficiency states and the associated pathology of these deficiencies [10]. Much evidence exists establishing the crucial role of B vitamins in immunity and it is well recognized that poor nutrition impacts significantly on immune cells [12]. Globally, chronic malnutrition presents as the main cause of immune deficiency and chronic diseases result from even marginal deficiencies [18]; vitamin B2, B6, and B9 are some of the B complex vitamins which are important contributors of nutritional support to the immune system. Deficiency of these can alter immune function considerably by disturbing nucleic acid production and protein synthesis, impeding immune cell activity, interfering with metabolic processes such as methylation and contributing to oxidative stress. Hyperhomocysteinemia is a result of ineffective methylation which triggers systemic and vascular inflammation and contributes to disease pathogenesis. Immune dysfunction at a cellular level can present as improper antigen presentation, disturbed cytokine production, unmodulated autoimmune responses [19,20], disruptions in immune cell function, and ineffective viral clearance [21]. Furthermore, vitamin B2 also plays a crucial role within the immune system because of its association with mucosal associated invariant T cells which play a significant role in autoimmune, inflammatory diseases and cancer [22].

The roles of B2, B6 and B9 in DNA and protein synthesis, cell maintenance and proliferation are a vital factor when looking at how deficiency states can affect the immune system. Both cell mediated, and humoral immunity is compromised in B vitamin deficiency states and the effects on immune cells are varied and numerous. Supplementation of B vitamins has been shown to improve the immune response in both animal and human models in many studies [23,24,25,26,27,28,29], however some studies have also reported that over supplementation can also adversely affect immune function [16,23,30].

B vitamin deficiencies can occur co-currently and seem to be particularly relevant in certain populations whereby dietary nutrition can limited by mitigating factors such as poor absorption, metabolism, illnesses and dietary restrictions. B vitamins have limited tissue storage and depending on the vitamin, can be depleted from the body as quickly as one week (B1, B2, B3, B5, B6, B7) to one month or a year after dietary deficiency (B9, B12) [31].

Numerous studies within the literature verify links between vitamin B deficiency and cancer through inflammatory processes. Vitamin B9 levels were found to be significantly lower and mean homocysteine levels significantly higher in newly diagnosed cancer patients than in aged match controls in one study, indicating that high homocysteine and low folate could be associated with lung cancer, although additional studies would be required to support these findings [32]. Another study involving breast cancer hypothesized that elevated homocysteine levels may have an effect on the methylation of some specific genes that control breast cancer initiation and progression. Breast cancer cell lines MCF-7 and MDA-MB-231 showed epigenetic modulations of RASS-F1 and BRACA1, as a result of elevated homocysteine levels. Elevated plasma homocysteine levels are also correlated with increased risk of colorectal cancer [33] whilst elevated levels of enzyme, PDXK, which facilitates in the conversion of pyridoxine (a vitamin B6 precursor) into pyridoxal-5′-phosphate the bioactive form of vitamin B6, indicated a good prognostic marker in patients suffering from non-small cell lung carcinoma [34].

In contrast however, some studies have associated an over-supplementation of some B vitamins in cancer progression. More recently it was noted that long-term supplementation of vitamin B12 in a total of 77,118 patients was associated with a 40% increased risk of lung cancer in males but not females in comparison with those who were not supplemented with B12. The study showed that B12 supplementation may in fact be harmful rather than chemo-preventative for lung cancer [35]. Another study noted that high levels of B12 are associated with a threefold risk of prostate cancer [36]. However, pooled data-based meta-analysis of vitamin B supplementation effects on the incidence of cancer showed that vitamin B supplementation did not have an effect on cancer incidence [37]. Conflicting evidence on B supplementation and cancer leads us to a justification of this projects proposition that B vitamin status and dose response is an important factor to explore.

B vitamin deficiencies can increase homocysteine levels which in turn can lead to oxidative stress, DNA damage and a chronic state of inflammation leading to epigenetic change and subsequent tumorigenesis [38]. Some B vitamins such as vitamin B6 have been indicated as anti-oxidant nutrients and as such, exert a protective role against inflammation and cancer progression [39,40,41].

Monocytes are circulatory blood cells, which differentiate into macrophages or dendritic cells at tissue sites. Macrophages and dendritic cells are specialized antigen presenting cells and process and present antigens to T cells, and secrete cytokines [42]. Histiocytic lymphoma is an aggressive non-Hodgkin’s lymphoma, a type of cancer that originates from cells of the immune system, specifically of monocyte pro-monocytic blast origin. The relationship between inflammation, carcinogenesis and vitamin B status is not completely understood and many of the mechanistic and molecular processes involved in this association are yet unresolved. The principal goal in this study was to determine the effect of supplementing un-differentiated pro-monocytic lymphoma cells with vitamin B2, B6 or B9 and we note altered effects on cell proliferation, migration, apoptosis, cytokines and inflammatory marker expression, as well expression of PD-L1.

## 2. Results

### 2.1. Vitamins B2, B6, B9 Inhibit Pro-Monocytic U937 Cell Proliferation

**Figure 1:**
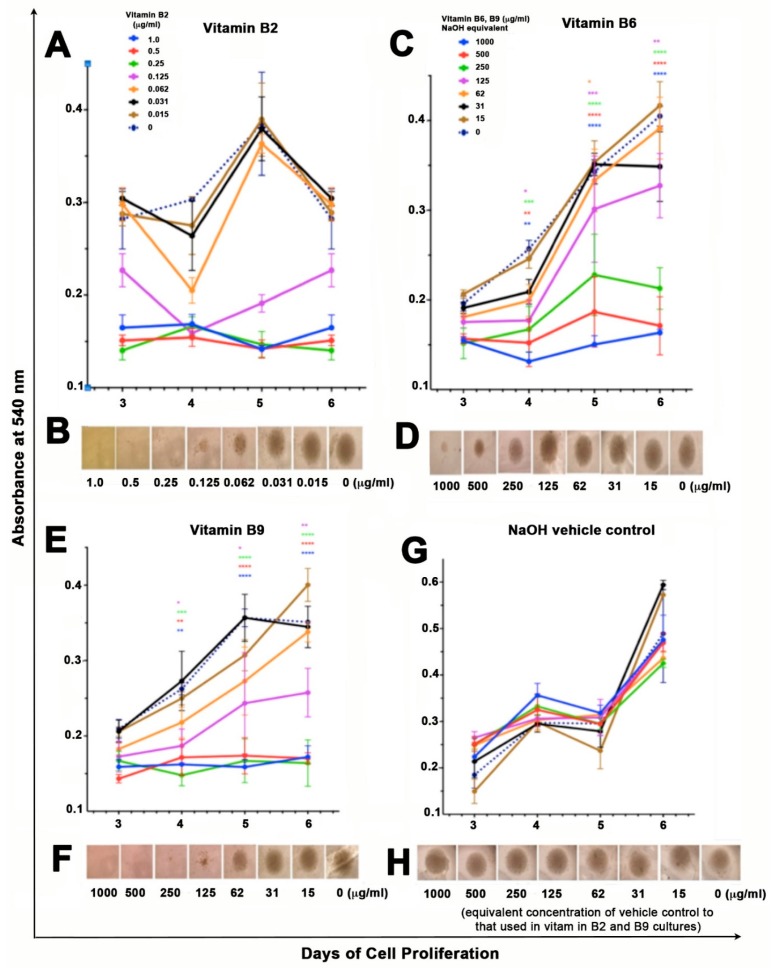
(**A**,**B**), Effect of vitamin B2 (riboflavin) (**C**,**D**), vitamin B6 (pyridoxine) (**E**,**F**), vitamin B9 (folic acid) (**G**,**H**), NaOH control on U937 cell proliferation. Cells were incubated with increasing doses of vitamin B for 6 days in 96 well U bottom plates and analyzed by MTT assay. Absorbance readings were taken at 540 nm to assess for cellular proliferation compared to control well (0 μg/mL). Significance was established at *p*, 0.05, two-way analysis of variance (ANOVA) followed by Tukey’s multiple comparisons test and marked with asterisk (* *p* < 0.05, ** *p* < 0.01, *** *p* < 0.001, **** *p* < 0.0001). Cells were viewed under an IX81 Olympus microscope at 4x magnification and photos taken at each concentration and control NaOH on day 6 of culture.

The effects of vitamin B2, B6 and B9 on cellular proliferation of U937 pro-monocytic cells was assessed using MTT (3-(4,5-Dimethylthiazol-2-yl)-2,5-diphenyltetrazolium bromide, a tetrazole) proliferation assay. All experiments were repeated at least 5 times in triplicate wells. Doses of 0.062 μg/mL, 0.031 μg/mL and 0.015 μg/mL of vitamin B2 (riboflavin), cell proliferation was similar to that of control cells whereas as 0.125 μg/mL inhibition of cell proliferation was noted (*p* < 0.05). Significant anti-proliferative effects were noted at doses 0.250 μg/mL–1.0 μg/mL (*p* < 0.0001) (Figure 1A). These findings were verified by well photos (Figure 1B).

Incubation of U937 cells with vitamin B6 (pyridoxine) showed no anti-proliferative effects on day 3 however on days 4–6 the anti-proliferative effects increased significantly in a dose dependent manner. On day 6, 1000 μg/mL, 500 μg/mL, and 250 μg/mL, showed the most inhibition (*p* < 0.0001), followed by less but significant inhibition at 125 μg/mL (*p* < 0.01). No anti-proliferative effects of riboflavin at 15–62 μg/mL were noted (Figure 1C,D).

At high doses of vitamin B9 (folic acid; 250–1000 μg/mL) significant inhibition of cell proliferation was noted on day 4 (*p* < 0.01) and days 5 and 6 (*p* < 0.0001). Although there was a trend of lower proliferation on day 3, this was not significant (Figure 1E). At 125 μg/mL of folic acid concentration there was less proliferation, but significant anti-proliferative effects were noted on days 3–6 (*p* < 0.05). The anti-proliferative effects were specific to folic acid as the corresponding NaOH vehicle control concentrations did not have an effect on cell proliferation (Figure 1E,G) These findings were confirmed by well images (Figure 1F,H).

### 2.2. Vitamin B Does not Induce Apoptosis or Cell Death

**Figure 2:**
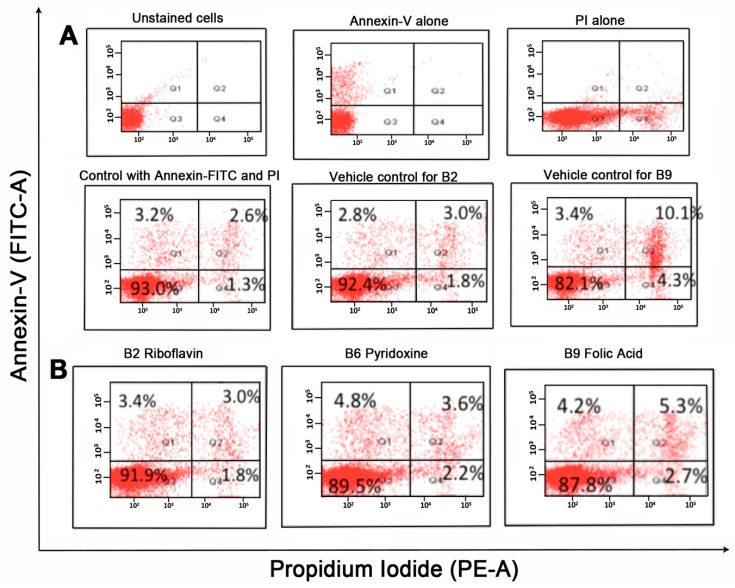
Annexin V-FITC/propridium iodide (PI) staining of undifferentiated U937 cells incubated with vitamin B. 1 × 10^6^ of U937 cells treated with 0.25 μg/mL of B2 and 250 μg/mL of vitamin B6 and B9 for 72 h were used for analysis. Resuspended cells were incubated with Annexin V-FITC at 1:1000 for 15 min in the dark. PI at 0.5 μg/mL was used as a counterstain to differentiate necrotic/dead cells from apoptotic cells. Shown in the figure are (**A**) controls, (**B**) vitamin B samples.

To determine whether the anti-proliferative and anti-migratory effects of vitamin B2, B6 and B9 were due to apoptosis or cell death, annexin-v assay was used which utilizes flow cytometry assay. Quadrants were set based on untreated control cells with either propridium iodide (PI) or FITC alone, or PI/FITC control staining (Figure 2). Q1 corresponds to early apoptosis (Annexin V FITC^+/^PI^−^) Q2 corresponds to dead cells by apoptosis (Annexin V FITC^+^/PI^+^), Q3 corresponds to live cells and non-apoptotic (Annexin V FITC^−^/PI^−^), Q4 demonstrates dead cells by necrosis or apoptosis (Annexin V FITC^−^/PI^+^). Control non-vitamin B treated cells were mostly viable 93%) and showing background levels of dead cells (Figure 2). The addition of vitamin B2, B6 and B9 250 μg/mL showed similar live/dead cell distribution as control, hence no evidence of apoptosis or death by necrosis is noted (Figure 2). Likewise, vitamin B2 and its vehicle control NaOH showed similar % of cell populations in each quadrant. Data for the 3-day vitamin B treatment is shown; treatment for 6 days showed similar effects (not shown).

### 2.3. Vitamin B2, B6, B9 Inhibits Cell Migration of Pro-Monocytic Cells

**Figure 3:**
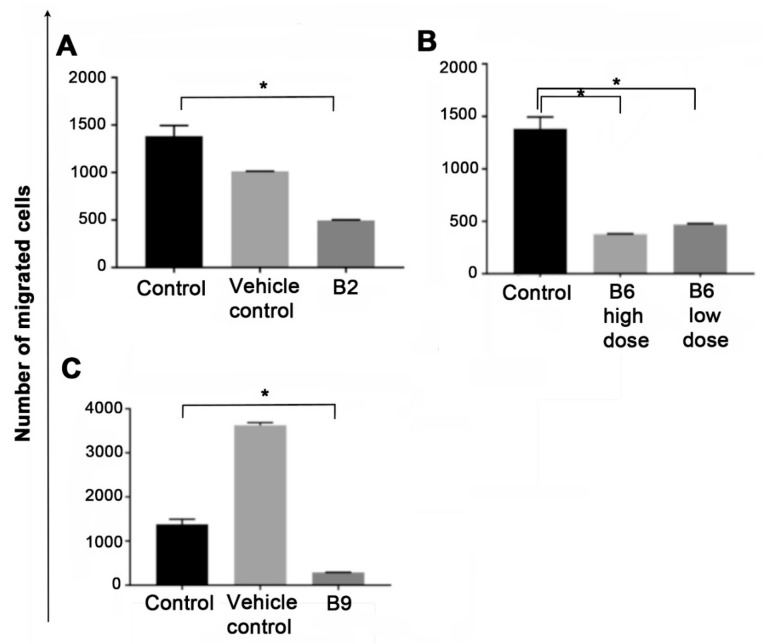
Effect on cell migration of pro-monocytic cells in the presence of (**A**) vitamin B2 (riboflavin), (**B**) vitamin B6 (pyridoxine) and (**C**) vitamin B9 (folic acid) using Boyden chamber assay. Data presented as mean +/− standard error of the mean of duplicate wells. Excel and Prism excel (Graph Pad Software, La Jolla, CA, USA) were used to aid in the statistical analysis using students *t*-Test and * *p* < 0.05 was considered significant.

Cell migration is evaluated via a number of different techniques such as microfluidic assays, scratch assays and cell-exclusion zone assays. However, the boyden chamber assay is the most widely accepted cell migration assay [39]. U937 pro-monocytic lymphoma cells were added inside the chamber and allowed to migrate through the porous membrane for 20–22 h. The number of cells that had migrated through the membrane were stained and counted using a light microscope [39]. Vitamin B2 (0.125 μg/mL), significantly reduced the number of cells migrating through the membrane (*p* < 0.5). Similarly, B6 (125 μg/mL, *p* < 0.05) and (250 μg/mL, *p* < 0.05), and B9 (125 μg/mL, *p* < 0.05), showed inhibition of cell migration (Figure 3). These data correspond to the anti-proliferative effects exhibited by vitamin B2, B6 and B9.

### 2.4. Vitamin B2, B6 and B9 Decrease Expression of PD-L1

**Figure 4:**
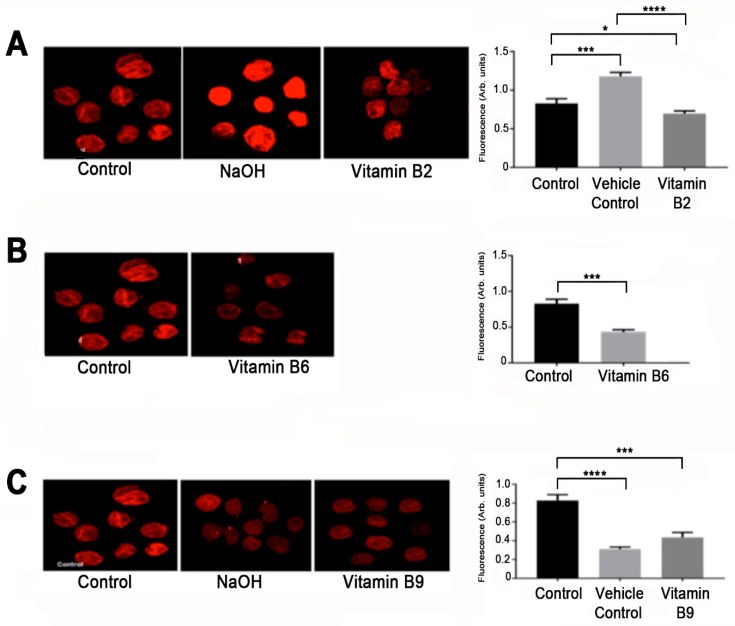
Expression of PD-L1 as assessed by confocal imaging on U937 cells in the presence of (**A**) vitamin B2 at 0.125 μg/mL and vitamin B2 vehicle control (NaOH) and untreated control. (**B**) Vitamin B6 at 125 μg/mL and untreated control, and, (**C**) vitamin B9 at 125 μg/mL and untreated control. (* *p* < 0.05, *** *p* < 0.001, **** *p* < 0.0001

Programmed death Ligand 1 (PD-L1) is an important checkpoint marker expressed by monocytes, macrophages, epithelial cells and cancer cells [40]. Expression of PD-L1 on cancer cells, has an immune suppression mechanism where cancer cells escape lysis by activated CD8^+^ T cells. PD-L1 is commonly overexpressed on tumor cells and aids in their invasiveness [41]. Confocal microscopy was used to assess the effect of B vitamins on PD-L1 expression. U937 cells cultured with vitamin B2, B6 or B9 showed reduced expression of PD-L1 compared to control (*p* < 0.05) and to NaOH vehicle control (*p* < 0.0001) (Figure 4). The number of tumor cells expressing PD-L1 were counted and fluorescence in arbitrary units was measured. Excel and Prism excel (Graph Pad Software, La Jolla, CA, USA) were used to aid in the statistical analysis and *p* < 0.05 was considered significant.

### 2.5. Vitamin B2, B6 and B9 Induce an Anti-Tumorigenic Cytokine Profile

**Figure 5:**
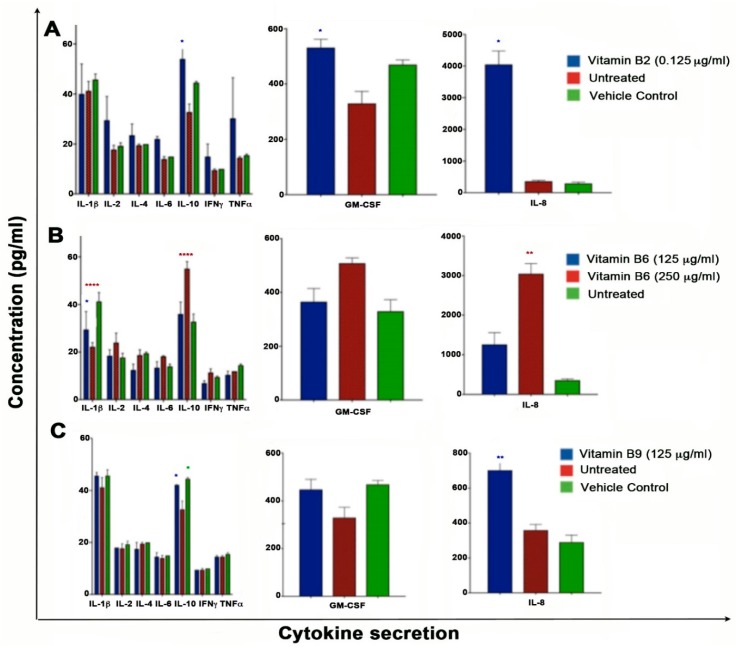
U937 cells were treated with (**A**) 0.125 μg/mL of vitamin B2 and NaOH vehicle control, (**B**) 125 μg/mL and 250 μg/mL of vitamin B6 and (**C**) 125 μg/mL of vitamin B9 and NaOH vehicle control for 3 days. Media and fresh vitamin B were replaced and cultured for a further 3 days. Supernatants were collected and cytokines secretion analyzed by bioplex. Significance in relation to untreated control is indicated by Asterisks (* *p* < 0.05, ** *p* < 0.01, **** *p* < 0.0001). Bioplex data for IL-1β, IL-2, Il-4, IL-6, Il-8, Il-10, IFNγ, TNFα, and GM-CSF are shown.

A magnetic bead bioplex assay was used to evaluate the effect of vitamin B (B2, B6 and B9) on cytokine secretion of promonocytic lymphoma U937 cells. Results are shown for vitamin B2, B6 and B9 as they showed significant anti-proliferative and migratory effects. Vitamin B2 significantly increased the secretion of IL-10, IL-6 and granulocyte colony stimulating factor (GM-CSF) (*p* < 0.05) (Table 1, Figure 5). Vitamin B6 also induced high levels of IL-10 (*p* < 0.001) and IL-8 (*p* < 0.001), and significant lower levels of IL-1β (*p* < 0.001) (Table 1, Figure 5). Vitamin B9 (folic acid) showed higher levels of IL-10 compared to control (*p* < 0.05) and significantly increased IL-8 secretion (*p* < 0.01). No other cytokines measured significance. It is clear that B2, B6 and B9 significantly increase IL-10 and IL-8 cytokine secretion by U937 cells. B6 also decreases IL-1β and B2 increases GM-CSF levels (Table 1, Figure 5).

## 3. Discussion

Vitamin B2 (riboflavin), B6 (pyridoxine), and B9 (folic acid) exert anti-proliferative and anti-migratory properties to U937 cells in a dose-dependent manner. The cells grown in the presence of either vitamin B2, B6 and B9 are healthy and viable, and only fewer cells grow under normal culture conditions. In fact, in apoptosis assays using annexin-V/PI staining, none of the B vitamins (B2, B6 and B9) induced apoptosis or cell death, suggesting that these B vitamins exert anti-proliferative/migratory properties. Further research is required to understand the mechanism behind these properties. Vitamin B2, is associated with energy production, anti-oxidant protection and homocysteine metabolism. Vitamin B2 deficiency is associated with growth retardation, anaemia, neurodegeneration and even certain cancers [43]. In fact, riboflavin deficiency in HepG2 cells was recently shown to promote cell proliferation and reduce cell viability [44]. In addition, vitamin B2 deficiency exacerbates iron deficiency and increases gastrointestinal cell crypt proliferation [45]. Conversely, the addition of riboflavin to lung cancer cell lines, leads to increased cell proliferation suggesting that riboflavin may promote the progression of lung cancer [46]. A vitamin B6 analogue, B6PR, at low doses cultured with the HUT78 cancer cell line has been shown to suppress cell proliferation and at high doses induces selective cell death [47]. Likewise, pyridoxal phosphate (a bioactive form of pyridoxine) inhibits rat pituitary adenoma cell lines even though the anti-proliferative effects were due to the apoptosis of cells [48]. In mice bearing colorectal cancer, supplementation of vitamin B6 reduces the number of tumors, cell proliferation and induces the expression of *c-myc* and *c-fos* proteins [49]. Similar to our findings, pyridoxine did not induce apoptosis of colon cancer cells, thus pyridoxine suppresses colon tumorigenesis by reducing cell proliferation [49]. Conversely, vitamin B6 supplementation into mice did not inhibit cell proliferation of glial cells, but rather promoted cell proliferation [50]. Furthermore, culture of colon cancer cell line, COLO-205, with folic acid (vitamin B9) inhibited cell proliferation via G0/G1 cell cycle arrest and through activation of c-SRC mediated pathway and increased levels of cyclin-dependent kinase inhibitor 1A, 1B and tumor protein p53 [51]. In addition, vitamin B9 supplementation to HCT116 and Caco-2 colon cancer cell lines reduced TGFβ secretion, induced cancer cell proliferation, and reduced tyrosine kinase activity and epidermal growth factor receptor expression [52]. The anti-proliferative and anti-migratory properties noted herein with vitamin B2, B6 and B9 are in accordance with previous published studies.

The potential mechanisms underlying the anti-proliferative and anti-migratory effects of vitamin B2, B6 and B9 may include, angiogenesis, altered cytokine secretion, altered PD-L1 expression, oxidative stress and nitric oxide synthesis. In fact, vitamin B2, B6 and B9 all increased the secretion of IL-8 and IL-10 by U937 cells compared to controls, with B2 additionally increasing GM-CSF and B6 additionally decreasing IL-1β. IL-8 an immune chemotaxis cytokine/chemokine correlates with angiogenesis, tumorigenicity, cell proliferation, invasiveness, metastasis and its expression on cancer cells is linked to poor prognosis [53,54]. Indeed, it has been shown that IL-8 increases proliferation of pancreatic and breast cancer cell lines [55,56]. Based on this information, it is not clear why IL-8 is upregulated by U937 cells in the presence of vitamin B2, B6, and B9 and the role of IL-8 expression by U937 cells is warranted. However, given that IL-8 is highly secreted by activated macrophages, it may be likely that the undifferentiated U937 cells are behaving more like macrophage cells rather than pro-monocytic blast cell lymphoma and hence indicating an activation state of macrophages. Furthermore, IL-10 was also upregulated in the presence of vitamin B2, B6 and B9. IL-10 is a potent anti-inflammatory (deactivates macrophages and monocytes) and immunosuppressive (inhibits antigen presenting cells and T cells) cytokine. On the other hand, IL-10 also exerts immuno-stimulatory effects, in particular to B cells, NK cells and CD8+ T cells [57]. Administration of recombinant (r), rIL-10 into cancer cells in vivo results in tumor rejection [57], mainly due to its ability to stimulate CD8+ T cells. Indeed, PEGylated rIL-10 (PEG-rIL-10; AM0010) increases tumor infiltrating CD8+ T cells four-fold in vivo, and results in tumor rejection and long-term protection [42]. As such, rIL-10 has recently surfaced as a treatment for intra-tumoral injection in cancer patients. In a dose escalation phase 1/1b trial of AM0010 in 14 different cancer types rIL-10 activated immune cells (CD8+ T cells), increased pro-inflammatory cytokines, decreased TGFβ, and induced partial and complete clinical responses [58]. At the 12th European International Kidney Cancer Symposium in Germany (21–22 April 2017) and ARMO BioSciences’ press release, it was announced that AM0010 in combination with checkpoint inhibitors induces better objective response rates and disease control of renal cell carcinoma patients in a phase 2 trial [59]. A phase III clinical trial (ClinicalTrials.gov identifier: NCT02923921) in combination with FOLFOX (folinic acid, 5-fluorouracil, oxaliplatin) chemotherapy is being conducted in patients with metastatic pancreatic carcinoma. Given the anti-tumor properties of rIL-10, the increased secretion of IL-10 by U937 cells in the presence of vitamin B2, B6 and B9 is likely to be one of the contributing factors to the anti-proliferative/anti-migratory effects noted herein. Moreover, vitamin B2 in addition increased the secretion of GM-CSF. GM-CSF is involved in proliferation, differentiation and function of myeloid-derived cells. Although GM-CSF has growth-promoting properties, it was noted over 30 years ago that GM-CSF inhibited the proliferation of U937 cells due to the activation of TNFα [60]. Consequently, rGM-CSF has been shown to increase the number of type-1 dendritic cells and is used in patients with cancer either on its own as an immune stimulant or as a vaccine adjuvant leading to anti-tumor responses [61,62]. Hence, the activation of GM-CSF by U937 cells in the presence of vitamin B2, is likely to contribute to the anti-proliferative effects noted. Finally, vitamin B6 reduced expression levels of IL-1β by U937 cells. IL-1β is known to promote tumor proliferation, angiogenesis, metastasis, in a number of cancers [63]. In fact, in IL-1 knockout mice, the growth of murine melanoma cell line was significantly reduced with no lung metastasis compared to wild-type mice [64]. As such, anti-IL-1β monoclonal antibodies (i.e., canakinumab) and IL-1β blockers (i.e., rilonacept) have been developed and show reduced cell proliferation, angiogenesis and metastasis of cancer cells in mice and in humans [65,66]. Given the tumor-promoting properties of IL-1β, the consequent reduced expression levels IL-1β by U937 cells in the presence of vitamin B6, is suggestive that this decrease may be a contributing factor to the anti-proliferative effects noted by vitamin B6.

To further gain insights into the mechanisms involved in anti-proliferative and anti-migratory effects of vitamin B2, B6 and B9, the expression levels of vascular endothelial growth factor-A (VEGF-A) and PD-L1 were determined. VEGF-A is a signal protein produced by cells to promote the formation of new blood vessels. Cancer cells express VEGF in order to grow (proliferate) and metastasize [67]. However, there was no significant alteration in intracellular expression levels of VEGF-A by U937 cells in the presence of vitamin B2, B6 and B9 (data not shown). As no major changes were noted to VEGF-A, the expression levels of VEGF isoforms (i.e., VEGF_121_, VEGF_145_, VEGF_165_, VEGF_189_, VEGF_206_) would give a more comprehensive analysis of the data; in addition to determining the levels of hypoxia-inducible factor (HIF) as this stimulates the release of VEGF. In addition, the expression of matrix-metalloporteinases-2, -9, involved in angiogenesis, cell proliferation and metastasis would build upon the mechanisms of action of vitamin B2, B6 and B9 on U937 cells.

PD-L1 was significantly downregulated in the presence of vitamin B2, B6 and B9. PD-L1 is a protein expressed on the surface of immune and non-immune cells. It serves as an immune checkpoint marker where it binds to its receptor PD-1 expressed on activated T cells leading to immune suppression [40,68,69,70,71]. Its increased expression on tumor cells leads to tumor progression (proliferation) and metastasis, and its expression is dependent on TNFα, IL-1β, IFNγ via toll-like receptors (TLRs) [72]. In fact, ovarian (ID8) and melanoma (B16) cell lines were transformed to express high levels of PD-L1 or PD-L1 was silenced; PD-L1 promoted cell proliferation in vitro and in vivo, whereas the silenced PD-L1 tumor cells resulted in slower proliferation rates in vitro and tumor load in mice [73]. Thus, the decreased expression levels of PD-L1 in the presence of vitamin B2, B6 and B9 corresponds to the reduced proliferation of U937 cells that was noted.

There are a few limitations in this study that could be addressed with further research. The first of these is that only one cell line was used. Other monocyte/macrophage cell lines should be used in repeat experiments to confirm the findings, for instance cell lines such as, THP-1, ML-2, HL-60, Mono mac6, RAW264.7, as well as dendritic cell lines T0525, JAWS II, KG-1 or MUTZ-3. A second limitation is that only a 9-plex bioplex assay kit was used. More information may be gathered by using, in repeat experiments, a 33-plex bioplex assay kit which would measure a wider array of cytokines as well as chemokines. Furthermore, in relation to length of cellular incubation, only one time point was used from the collection of supernatants for cytokine analysis. Other time points that were significant from the proliferation assays could also be used to present a more in depth analysis.

## 4. Materials and Methods

### 4.1. Cell Culture

The U937 cell line was isolated from a 37-year-old male patient with histiocytic lymphoma. U937 cells are commonly used to study the behavior and differentiation of monocytes. They are able to be differentiated into monocyte/macrophages following stimulation with certain stimulants such as, vitamin D3 [42]. U937 cells were obtained from Monash University Department of Immunology. U937 cells are immortal and allow for multiple passages without transformation; however, we ensured to keep the number of passages to a minimum. U937 cells were cultured in RPMI 1640 media supplemented with 2 nm. L-glutamine (Sigma-Aldrich, St Louis, MO, USA), 100 U/mL penicillin, 100 µg/mL streptomycin (Sigma-Aldrich, St Louis, MO, USA) and 10% heat-inactivated foetal bovine serum (FBS; Sigma-Aldrich, St Louis, MO, USA) at 37 °C and 5% CO_2_. Culture media was changed every 3–4 days and cells were passaged accordingly. Once 80%–90% confluent, cells were used in experiments.

### 4.2. Preparation of Vitamin B Stocks

A stock solution of each Vitamin B was freshly prepared according to the manufacturer’s instructions (Sigma, VIC, Australia) in either phosphate buffered saline (PBS) (Vitamin B6) or sodium hydroxide (NaOH—Vitamin B2, B9). The corresponding concentration of vehicle control NaOH was included. Each vitamin stock was filtered using 0.2-micron filters and further dilutions were made to 2 mg/mL in RPMI-1640 media, to allow for serial dilution of the working concentrations.

### 4.3. Proliferation of Pro-Monocytic Cells Using MTT

MTT (3-(4,5-Dimethylthiazol-2-yl)-2,5-diphenyltetrazolium bromide, a tetrazole) forms purple formazan in the mitochondria of living cells in the reduction reaction, which takes place only when mitochondrial enzymes are active and, therefore, conversion can be directly related to the number of viable cells [74]. The absorbance of the purple solution can be quantified by measuring a wavelength by a spectrophotometer. The amount of purple formazan produced by cells treated with vitamin B, (vitamin B2, B6, B9) with the amount of formazan produced by untreated cells or control solvent, determines the amount of cell death. Higher number of viable cells results in a greater amount of MTT formazan formation which triggers an increase in absorbance. U bottom 96-well plates (1 plate for each day of incubation) were seeded with 100 μL of titrated concentrations of vitamin B (1000 μg/mL, 500 μg/mL, 250 μg/mL, 125 μg/mL, 62.5 μg/mL, 31.25 μg/mL, 15.6 μg/mL and untreated control 0 μg/mL) and 100 μL of cell suspension (at density of 1 × 10^4^ cells/well) in triplicate for vitamins B6 and B9 and 1 μg/mL, 0.5 μg/mL, 0.25 μg/mL, 0.125 μg/mL, 0.62 μg/mL, 0.31 μg/mL, 0.15 μg/mL and untreated control 0 μg/mL for vitamin B2). Untreated cells and vehicle control wells corresponding to each dose of NaOH in the vitamin B2 and B9 wells were also used. The plates were incubated at 37 °C and 5% CO_2_ for three days. On day three of incubation, half the well volume was removed and replaced with fresh media/Vitamin B. MTT assay was performed on days 3, 4, 5 and 6 of incubation to assess cellular proliferation.

Cellular proliferation was assessed using a spectrophotometer (Bio-Rad microplate reader, 6.0) using wavelength 570 nm. Three-five independent experiments were conducted in triplicates. Supernatants from each day were transferred to cryovials and stored at −80 °C for later use of cytokine analysis using the Bioplex system.

### 4.4. Visual Assessment of Cell Proliferation

On incubation days, 3, 4, 5 and 6 plates were viewed under an IX81 Olympus microscope and photos were taken of one triplicate of each concentration at 4× magnification. Photos and figures were then collated for each vitamin, day and concentration.

### 4.5. Cell Migration Assays

Cells were treated with vitamin B2 0.125 μg/mL, B6 125 μg/mL and 250 μg/mL or B9 125 μg/mL, (corresponding with IC-50 amounts for 3 days after which fresh media and vitamin B added and allowed to incubate a further 3 days. Controls included untreated and NaOH at corresponding doses to the B vitamins dissolved in NaOH. Cell migration was performed using the Boyden chamber assay 8-µm pore size membrane filter inserts in 24-well tissue culture plates. The cells were trypsinized and re-suspended in serum-free RPMI-1640 media at the density of 2 × 10^5^ cells/mL. A total of 200 µL of cells suspension was seeded in the upper chamber of the trans wells, and 600 µL of media into the lower chamber. The chambers were incubated at 37 °C in a humid atmosphere with 5% CO_2_. After 20–22 h, the non-migrating cells on the upper surface of the insert were removed and the cells that migrated to the underside of the membrane were counted using a light microscope. Excel and Prism excel (Graph Pad Software, La Jolla, CA, USA) were used to aid in the statistical analysis using Student’s *t*-test and *p* < 0.05 was considered significant.

### 4.6. Apoptosis Assay

#### 4.6.1. Annexin V FITC/Propridium Iodide (PI)

Changes in the plasma membrane and loss of membrane asymmetry are the amongst the earliest indications of apoptosis. The translocation of the membrane phospholipid phosphatidylserine (PS) from the inner to the outer membrane exposes PS to the external cellular environment. Annexin V, having high affinity for PS binds to PS presenting cells and signals the beginning of membrane degradation seen in the last stages of cell death caused by apoptosis or necrosis [75]. Using an accompanying dye such as FITC helps to signal cells which are in the early stages of apoptosis. Viable non-apoptotic cells are Annexin V negative and PI negative. Cell dead by apoptosis are Annexin V positive and PI positive. Cells in early apoptosis are Annexin V positive and propidium iodide negative while cells dead by either apoptosis or necrosis are Annexin V negative and PI positive.

#### 4.6.2. Sample Preparation and Flow Cytometry

U937 cells (100 μL) at 1 × 10^6^ cells/mL were co-cultured with 100 μL of vitamin B/RPMI media in 96 well plates for 3 days and 1 mL of U937 cells in 9 mL of vitamin B/RPMI media for 6 days at the following concentrations. Vitamin B2-0.250 μg/mL and * 0.95 μg/mL, B6-250 μg/mL and * 190 μg/mL, B9-250 μg/mL and * 120 μg/mL, NaOH-6.2 mM and 250 μM vehicle controls corresponding to the amount of NaOH within the vitamin B well) or culture media alone. * Amounts with asterisk coincided with IC-50 amounts calculated from previous MTT assay.

U937 cells, were harvested by centrifugation at 1200 rpm for 5 min at room temperature. The supernatant was discarded, and the cells were washed twice in 200 μL of FACS buffer (PBS, 1 mM EDTA, 2% foetal calf serum and 0.1% sodium azide) by pipetting. Cells were then re-suspended in 100 μL per well of Annexin V binding buffer with FITC Annexin V (BioLegend, San Diego, CA, USA) at 1:1000 and 0.5 μg/mL of PI. Wells containing untreated, FITC and PI, PI alone, FITC alone were set up as controls. Samples were transferred to FACS tubes and data were collected using BD FACS canto^TM^ II cell analyser at medium setting; 30,000 events were collected. Cells were analysed using BD FACS diva software. Results were displayed as percentages in quadrant boxes corresponding to the data in Figure 2A.

### 4.7. Bioplex Cytokine Assay

The Bioplex human cytokine immunoassay is a highly sensitive and reproducible magnetic bead-based assay which allows accurate measurement of low levels of human cytokines. The Bioplex cytokine assay uses 8 μm magnetic beads coated with antibodies against an array of cytokines. Cytokine assays were performed using a bead-based multiplex immunoassay (MIA, 9 Bio-Plex Panel B, BioRad Laboratories Inc., Melbourne, VIC, Australia) that included the cytokines IL-1β, IL-2, IL-4, IL-6, IL-8, IL-10, IFNγ, TNFα, GM-CSF. Supernatant samples from day 6 of vitamin B culture were run neat (day 6 was chosen as this is the day that showed the most effect of anti-proliferation). In addition, 24 h-time point was used to collect supernatants for differentiated U937 cells stimulated with vitamin B and cytokines assessed. Standard low photomultiplier tube settings were prepared with “blank” negative controls in duplicate. Ninety-six-well plates were coated with beads, followed by the addition of samples and standards and detection antibodies, then streptavidin-phytoerythrin as per the manufacturer’s instructions. The beads were re-suspended and the fluorescence output read and calculated on the Bio-Plex array reader (Bio-rad, Melbourne, VIC, Australia). Statistical analysis of data included the mean and standard deviation (SD), as well as a two-way analysis of variance (ANOVA) followed by a Sidaks multiple comparison test using GraphPad Prism^TM^ (Graphpad Software, San Diego, CA, USA). Significance was defined as *p* < 0.05.

### 4.8. PD-L1 Expression and Confocal Microscopy

Programmed death ligand 1 (PD-L1) is expressed by cancer cells as a way to escape T cell immune responses. Recently, it was shown that there is a relationship between the expression of PD-L1 with proliferation and invasion of cells [41], hence, the expression of PD-L1 on U937 cells with or without vitamin B treatment was determined. Confocal microscopy was used in order to visually determine its expression on U937 cells. U937 cells were treated with vitamin B2 (0.125 μg/mL), B6 (125 μg/mL and 250 μg/mL), B9 (125 μg/mL) for 6 days. Controls included untreated cells and NaOH at corresponding doses to the B vitamins dissolved in NaOH. U937 treated cells (100 μL) at 5 × 10^5^ cells/mL were added to 96 well round bottom plates and washed 2 × 5 min with PBS. Cells were fixed with 4% paraformaldehyde for 10 min. Cells were permeabilized for 15 min in 0.1% Triton X-100/PBS. Non-specific binding was blocked using 10% donkey serum for 1 h at room temperature. Plates were then washed twice for 4 min with 0.1% Triton/PBS. Cells were labelled with primary monoclonal antibody PD-L1 (Abcam, Melbourne, Australia, ab210931) at 1:500 and incubated for 2 h at room temperature. Cells were washed twice in 0.1% Triton X-100/PBS and incubated at room temperature in the dark for 2 h. Secondary antibody Alexa Fluor 647-conjugated donkey anti-mouse at 1:250 dilution (Abacus, JI715605150). After two washes with 0.1% Triton X-100/PBS, cells were incubated for 2 min with DAPI washed and 2 small drops of cells were placed onto slides and left to dry. Slides were mounted with fluorescence mounting media, cover slip applied, over the cells and clear nail polish applied around the perimeter of the coverslip. Slides were labelled and left to dry wrapped in tin foil in the dark overnight.

Images were captured on a Nikon Eclipse Ti multichannel confocal laser scanning system (Nikon, Tokyo, Japan). Z-series images were acquired at a nominal thickness of 0.5 μm (512 × 512 pixels). The numbers of U937 cells expressing PD-L1 markers were counted within eight randomly captured images (total area size 2 mm^2^) per preparation at ×20 magnification. Image J software (National Institute of Health, Bethesda, MD, USA) was employed to convert images from RGB to greyscale 8 bit then to binary; particles were then analyzed to obtain the percentage area of immunoreactivity. Excel and Prism excel (Graph Pad Software, La Jolla, CA, USA) were used to aid in the statistical analysis and *p* < 0.05 was considered significant.

### 4.9. Data Analysis Using Excel and Prism Programs

Statistical analysis of MTT proliferation assays was performed using 2way ANOVA and Tukeys multiple comparisons test. The mean of triplicate wells was considered significantly different when compared to vehicle control groups if *p* < 0.05 in proliferation assays.

## 5. Conclusions

In conclusion, it is clear that vitamin B2, B6 and B9 inhibits pro-monocytic cell proliferation and migration which is not due to apoptosis or cell death. It is not clear what role IL-8 plays in the anti-proliferative and anti-migratory effects, as it is generally believed that IL-8 is associated with increased proliferation, migration and angiogenesis of cancer cells; the mechanism associated with increased levels of IL-8 needs to be further elucidated. However, collectively, vitamin B2, B6 and B9 increased IL-10, GM-CSF and decreased IL-1β cytokine levels which are in accordance with reduced cell proliferation and anti-tumorigenic properties based on other studies in the literature. In addition, the decreased expression levels of PD-L1 supports the reduced proliferation and migration of U937 cells. The data give insights into the anti-tumorigenic properties of vitamin B2, B6 and B9 and could form the basis for future studies in using vitamin B supplementation to reduce cancer cell growth in vivo in animal models and in humans. Results of this study suggest that B2 B6 and B9 could be recognized as potential anti-tumorigenic nutrients, although subsequent studies would need to be completed to gain more insight into the mechanisms behind these anti-tumor effects. Future studies may further illuminate some of these mechanisms by observing changes in gene expression of key immune cells.

## Figures and Tables

**Table 1:** Summary of cytokine secretion by promonocytic U937 cell line.

Vitamin	Cytokine								
	IL-1β	IL-2	IL4	IL-6	IL-8	IL-10	IFNγ	TNFα	GM-CSF
**B2**	**-**	**-**	**-**	**-**	**increase**	**increase**	**-**	**-**	**increase**
**B6**	**decrease**	**-**	**-**	**-**	**increase**	**increase**	**-**	**-**	**-**
**B9**	**-**	**-**	**-**	**-**	**increase**	**increase**	**-**	**-**	**-**

## References

[1] Rakoff-Nahoum S. (2006). Why cancer and inflammation?. Yale J. Biol. Med..

[2] Coussens L.M., Werb Z. (2002). Inflammation and cancer. Nature.

[3] Itzkowitz S.H., Yio X. (2004). Inflammation and cancer IV. Colorectal cancer in inflammatory bowel disease: The role of inflammation. Am. J. Physiol. Gastrointest Liver Physiol..

[4] Chang H.C., Yen A.M., Fann J.C., Chiu S.Y., Liao C.S., Chen H.H., Yang K.C., Chen L.S., Lin Y.M. (2015). Irritable bowel syndrome and the incidence of colorectal neoplasia: A prospective cohort study with community-based screened population in Taiwan. Br. J. Cancer.

[5] Moody G.A., Jayanthi V., Probert C.S., Mac Kay H., Mayberry J.F. (1996). Long-term therapy with sulphasalazine protects against colorectal cancer in ulcerative colitis: A retrospective study of colorectal cancer risk and compliance with treatment in Leicestershire. Eur. J. Gastroenterol. Hepatol..

[6] Haverkamp J., Charbonneau B., Ratliff T.L. (2008). Prostate inflammation and its potential impact on prostate cancer: A current review. J. Cell Biochem..

[7] Block T.M., Mehta A.S., Fimmel C.J., Jordan R. (2003). Molecular viral oncology of hepatocellular carcinoma. Oncogene.

[8] Allavena P., Garlanda C., Borrello M.G., Sica A., Mantovani A. (2008). Pathways connecting inflammation and cancer. Curr. Opin. Genet. Dev..

[9] Mikkelsen K., Apostolopoulos V. (2018). B Vitamins and Ageing. Sub Cell. Biochem..

[10] Mikkelsen K., Stojanovska L., Apostolopoulos V. (2016). The Effects of Vitamin B in Depression. Curr. Med. Chem..

[11] Mikkelsen K., Stojanovska L., Polenakovic M., Bosevski M., Apostolopoulos V. (2017). Exercise and mental health. Maturitas.

[12] Mikkelsen K., Stojanovska L., Prakash M., Apostolopoulos V. (2017). The effects of vitamin B on the immune/cytokine network and their involvement in depression. Maturitas.

[13] Mikkelsen K., Stojanovska L., Tangalakis K., Bosevski M., Apostolopoulos V. (2016). Cognitive decline: A vitamin B perspective. Maturitas.

[14] Cianciolo G., De Pascalis A., Di Lullo L., Ronco C., Zannini C., La Manna G. (2017). Folic Acid and Homocysteine in Chronic Kidney Disease and Cardiovascular Disease Progression: Which Comes First?. Cardiorenal. Med..

[15] Clarke R., Smith A.D., Jobst K.A., Refsum H., Sutton L., Ueland P.M. (1998). Folate, vitamin B12, and serum total homocysteine levels in confirmed Alzheimer disease. Arch. Neurol..

[16] Henry C.J., Nemkov T., Casas-Selves M., Bilousova G., Zaberezhnyy V., Higa K.C., Serkova N.J., Hansen K.C., D’Alessandro A., DeGregori J. (2017). Folate dietary insufficiency and folic acid supplementation similarly impair metabolism and compromise hematopoiesis. Haematologica.

[17] Mahmood L. (2014). The metabolic processes of folic acid and Vitamin B12 deficiency. J. Health Res. Rev..

[18] Schaible U.E., Kaufmann S.H. (2007). Malnutrition and infection: Complex mechanisms and global impacts. PLoS Med..

[19] Dargahi N., Johnson J., Donkor O., Vasiljevic T., Apostolopoulos V. (2019). Immunomodulatory effects of probiotics: Can they be used to treat allergies and autoimmune diseases?. Maturitas.

[20] Dargahi N., Katsara M., Tselios T., Androutsou M.E., de Courten M., Matsoukas J., Apostolopoulos V. (2017). Multiple Sclerosis: Immunopathology and Treatment Update. Brain Sci..

[21] Bayer A.L., Fraker C.A. (2017). The Folate Cycle As a Cause of Natural Killer Cell Dysfunction and Viral Etiology in Type 1 Diabetes. Front. Endocrinol..

[22] Kumar V., Ahmad A. (2018). Role of MAIT cells in the immunopathogenesis of inflammatory diseases: New players in old game. Int. Rev. Immunol..

[23] Adhikari P.M., Chowta M.N., Ramapuram J.T., Rao S.B., Udupa K., Acharya S.D. (2016). Effect of Vitamin B12 and folic acid supplementation on neuropsychiatric symptoms and immune response in HIV-positive patients. J. Neurosci. Rural Pract..

[24] Au-Yeung K.K., Yip J.C., Siow Y.L., O K. (2006). Folic acid inhibits homocysteine-induced superoxide anion production and nuclear factor kappa B activation in macrophages. Can. J. Physiol. Pharmacol..

[25] Fukuda S., Koyama H., Kondo K., Fujii H., Hirayama Y., Tabata T., Okamura M., Yamakawa T., Okada S., Hirata S. (2015). Effects of nutritional supplementation on fatigue, and autonomic and immune dysfunction in patients with end-stage renal disease: A randomized, double-blind, placebo-controlled, multicenter trial. PLoS ONE.

[26] Gross R.L., Reid J.V., Newberne P.M., Burgess B., Marston R., Hift W. (1975). Depressed cell-mediated immunity in megaloblastic anemia due to folic acid deficiency. Am. J. Clin. Nutr..

[27] Huang T., Li K., Asimi S., Chen Q., Li D. (2015). Effect of vitamin B-12 and n-3 polyunsaturated fatty acids on plasma homocysteine, ferritin, C-reaction protein, and other cardiovascular risk factors: A randomized controlled trial. Asia Pac. J. Clin. Nutr..

[28] Lewicki S., Lewicka A., Kalicki B., Klos A., Bertrandt J., Zdanowski R. (2014). The influence of vitamin B12 supplementation on the level of white blood cells and lymphocytes phenotype in rats fed a low-protein diet. Cent. Eur. J. Immunol.

[29] Scalabrino G., Corsi M.M., Veber D., Buccellato F.R., Pravettoni G., Manfridi A., Magni P. (2002). Cobalamin (vitamin B(12)) positively regulates interleukin-6 levels in rat cerebrospinal fluid. J. Neuroimmunol..

[30] Meadows D.N., Bahous R.H., Best A.F., Rozen R. (2015). High Dietary Folate in Mice Alters Immune Response and Reduces Survival after Malarial Infection. PLoS ONE.

[31] Sechi G., Sechi E., Fois C., Kumar N. (2016). Advances in clinical determinants and neurological manifestations of B vitamin deficiency in adults. Nutr. Rev..

[32] Tastekin D., Erturk K., Bozbey H.U., Olmuscelik O., Kiziltan H., Tuna S., Tas F. (2015). Plasma homocysteine, folate and vitamin B12 levels in patients with lung cancer. Exp. Oncol..

[33] Miller J.W., Beresford S.A., Neuhouser M.L., Cheng T.Y., Song X., Brown E.C., Zheng Y., Rodriguez B., Green R., Ulrich C.M. (2013). Homocysteine, cysteine, and risk of incident colorectal cancer in the Women’s Health Initiative observational cohort. Am. J. Clin. Nutr..

[34] Galluzzi L., Vacchelli E., Michels J., Garcia P., Kepp O., Senovilla L., Vitale I., Kroemer G. (2013). Effects of vitamin B6 metabolism on oncogenesis, tumor progression and therapeutic responses. Oncogene.

[35] Brasky T.M., White E., Chen C.L. (2017). Long-Term, Supplemental, One-Carbon Metabolism-Related Vitamin B Use in Relation to Lung Cancer Risk in the Vitamins and Lifestyle (VITAL) Cohort. J. Clin. Oncol..

[36] Hultdin J., Van Guelpen B., Bergh A., Hallmans G., Stattin P. (2005). Plasma folate, vitamin B12, and homocysteine and prostate cancer risk: A prospective study. Int. J. Cancer.

[37] Zhang S.L., Chen T.S., Ma C.Y., Meng Y.B., Zhang Y.F., Chen Y.W., Zhou Y.H. (2016). Effect of vitamin B supplementation on cancer incidence, death due to cancer, and total mortality: A PRISMA-compliant cumulative meta-analysis of randomized controlled trials. Medicine.

[38] Khanna R., Karki K., Pande D., Negi R., Khanna R.S. (2014). Inflammation, Free Radical Damage, Oxidative Stress and Cancer. Int. J. Inflamm. Cancer Integr. Ther..

[39] Chen H.C. (2005). Boyden chamber assay. Methods Mol. Biol..

[40] Kuol N., Stojanovska L., Nurgali K., Apostolopoulos V. (2017). The mechanisms tumor cells utilize to evade the host’s immune system. Maturitas.

[41] Xue S., Hu M., Li P., Ma J., Xie L., Teng F., Zhu Y., Fan B., Mu D., Yu J. (2017). Relationship between expression of PD-L1 and tumor angiogenesis, proliferation, and invasion in glioma. Oncotarget.

[42] Chanput W., Peters V., Wichers H.J. (2015). THP-1 and U937 Cells. The Impact of Food Bioactives on gut Health.

[43] Thakur K., Tomar S.K., Brahma B., De S. (2016). Screening of Riboflavin-Producing Lactobacilli by a Polymerase-Chain-Reaction-Based Approach and Microbiological Assay. J. Agric. Food Chem..

[44] Liu D., Ke Z., Luo J. (2017). Thiamine Deficiency and Neurodegeneration: The Interplay Among Oxidative Stress, Endoplasmic Reticulum Stress, and Autophagy. Mol. Neurobiol..

[45] Werner R., Manthey K.C., Griffin J.B., Zempleni J. (2005). HepG2 cells develop signs of riboflavin deficiency within 4 days of culture in riboflavin-deficient medium. J. Nutr. Biochem..

[46] Chen Y., Ma J., Wang F., Hu J., Cui A., Wei C., Yang Q., Li F. (2013). Amygdalin induces apoptosis in human cervical cancer cell line HeLa cells. Immunopharmacol. Immunotoxicol..

[47] Kesel A.J., Sonnenbichler I., Polborn K., Gurtler L., Klinkert W.E., Modolell M., Nussler A.K., Oberthur W. (1999). A new antioxidative vitamin B6 analogue modulates pathophysiological cell proliferation and damage. Bioorg. Med. Chem..

[48] Frasure-Smith N., Lesperance F. (2006). Depression and coronary artery disease. Herz.

[49] Komatsu S.I., Watanabe H., Oka T., Tsuge H., Nii H., Kato N. (2001). Vitamin B-6-supplemented diets compared with a low vitamin B-6 diet suppress azoxymethane-induced colon tumorigenesis in mice by reducing cell proliferation. J. Nutr..

[50] Yoo D.Y., Kim W., Kim D.W., Yoo K.Y., Chung J.Y., Youn H.Y., Yoon Y.S., Choi S.Y., Won M.H., Hwang I.K. (2011). Pyridoxine enhances cell proliferation and neuroblast differentiation by upregulating the GABAergic system in the mouse dentate gyrus. Neurochem. Res..

[51] Kuo C.T., Chang C., Lee W.S. (2015). Folic acid inhibits COLO-205 colon cancer cell proliferation through activating the FRalpha/c-SRC/ERK1/2/NFkappaB/TP53 pathway: In vitro and in vivo studies. Sci. Rep..

[52] Jaszewski R., Khan A., Sarkar F.H., Kucuk O., Tobi M., Zagnoon A., Dhar R., Kinzie J., Majumdar A.P. (1999). Folic acid inhibition of EGFR-mediated proliferation in human colon cancer cell lines. Am. J. Physiol..

[53] Seaton A., Scullin P., Maxwell P.J., Wilson C., Pettigrew J., Gallagher R., O’Sullivan J.M., Johnston P.G., Waugh D.J. (2008). Interleukin-8 signaling promotes androgen-independent proliferation of prostate cancer cells via induction of androgen receptor expression and activation. Carcinogenesis.

[54] Waugh D.J., Wilson C. (2008). The interleukin-8 pathway in cancer. Clin. Cancer Res. Off. J. Am. Assoc. Cancer Res..

[55] Chen L., Fan J., Chen H., Meng Z., Chen Z., Wang P., Liu L. (2014). The IL-8/CXCR1 axis is associated with cancer stem cell-like properties and correlates with clinical prognosis in human pancreatic cancer cases. Sci. Rep..

[56] Singh J.K., Simoes B.M., Howell S.J., Farnie G., Clarke R.B. (2013). Recent advances reveal IL-8 signaling as a potential key to targeting breast cancer stem cells. Breast Cancer Res. BCR.

[57] Goldman M., Velu T., Pretolani M. (1997). Interleukin-10: Actions and therapeutic potential. BioDrugs Clin. Immunother. Biopharm. Gene Ther..

[58] Naing A., Papadopoulos K.P., Autio K.A., Ott P.A., Patel M.R., Wong D.J., Falchook G.S., Pant S., Whiteside M., Rasco D.R. (2016). Safety, Antitumor Activity, and Immune Activation of Pegylated Recombinant Human Interleukin-10 (AM0010) in Patients With Advanced Solid Tumors. J. Clin. Oncol. Off. J. Am. Soc. Clin. Oncol..

[59] Meloa A. (2017). Latest Results Presented on ARMO’s Lead Immunotherapy Candidate, AM0010. https://immuno-oncologynews.com/2017/04/27/armo-clinical-data-am0010-immunotherapy/.

[60] Cannistra S.A., Rambaldi A., Spriggs D.R., Herrmann F., Kufe D., Griffin J.D. (1987). Human granulocyte-macrophage colony-stimulating factor induces expression of the tumor necrosis factor gene by the U937 cell line and by normal human monocytes. J. Clin. Investig..

[61] Arellano M., Lonial S. (2008). Clinical uses of GM-CSF, a critical appraisal and update. Biol. Targets Ther..

[62] Yan W.L., Shen K.Y., Tien C.Y., Chen Y.A., Liu S.J. (2017). Recent progress in GM-CSF-based cancer immunotherapy. Immunotherapy.

[63] Lewis A.M., Varghese S., Xu H., Alexander H.R. (2006). Interleukin-1 and cancer progression: The emerging role of interleukin-1 receptor antagonist as a novel therapeutic agent in cancer treatment. J. Transl. Med..

[64] Voronov E., Shouval D.S., Krelin Y., Cagnano E., Benharroch D., Iwakura Y., Dinarello C.A., Apte R.N. (2003). IL-1 is required for tumor invasiveness and angiogenesis. Proc. Natl. Acad. Sci. USA.

[65] De Mooij C.E.M., Netea M.G., van der Velden W., Blijlevens N.M.A. (2017). Targeting the interleukin-1 pathway in patients with hematological disorders. Blood.

[66] Dinarello C.A. (2010). Why not treat human cancer with interleukin-1 blockade?. Cancer Metastasis Rev..

[67] Mahecha A.M., Wang H. (2017). The influence of vascular endothelial growth factor-A and matrix metalloproteinase-2 and -9 in angiogenesis, metastasis, and prognosis of endometrial cancer. OncoTargets Ther..

[68] Kuol N., Stojanovska L., Apostolopoulos V., Nurgali K. (2018). Role of the Nervous System in Tumor Angiogenesis. Cancer Microenviron. Off. J. Int. Cancer Microenviron. Soc..

[69] Kuol N., Stojanovska L., Apostolopoulos V., Nurgali K. (2018). Role of the nervous system in cancer metastasis. J. Exp. Clin. Cancer Res. CR.

[70] Kuol N., Stojanovska L., Apostolopoulos V., Nurgali K. (2018). Crosstalk between cancer and the neuro-immune system. J. Neuroimmunol..

[71] Kuol N., Stojanovska L., Nurgali K., Apostolopoulos V. (2018). PD-1/PD-L1 in disease. Immunotherapy.

[72] Brogden K.A., Vali S., Abbasi T. (2016). PD-L1 is a diverse molecule regulating both tumor-intrinsic signaling and adaptive immunosuppression. Transl. Cancer Res..

[73] Clark C.A., Gupta H.B., Sareddy G., Pandeswara S., Lao S., Yuan B., Drerup J.M., Padron A., Conejo-Garcia J., Murthy K. (2016). Tumor-Intrinsic PD-L1 Signals Regulate Cell Growth, Pathogenesis, and Autophagy in Ovarian Cancer and Melanoma. Cancer Res..

[74] Riss T.L., Moravec R.A., Niles A.L., Duellman S., Benink H.A., Worzella T.J., Minor L., Sittampalam G.S., Coussens N.P., Brimacombe K., Grossman A., Arkin M., Auld D., Austin C., Baell J., Bejcek B., Chung T.D.Y. (2004). Cell Viability Assays. Assay Guidance Manual.

[75] Demchenko A.P. (2013). Beyond annexin V: Fluorescence response of cellular membranes to apoptosis. Cytotechnology.

